# Inclusion of Individuals With Neurodevelopmental Disorders in Norm-Referenced Language Assessments

**DOI:** 10.3389/fpsyg.2022.929433

**Published:** 2022-08-12

**Authors:** Susan J. Loveall, Marie Moore Channell, Laura J. Mattie, Alexandria E. Barkhimer

**Affiliations:** ^1^Department of Special Education and Communication Disorders, University of Nebraska–Lincoln, Lincoln, NE, United States; ^2^Department of Speech and Hearing Science, University of Illinois at Urbana-Champaign, Champaign, IL, United States

**Keywords:** language assessment, neurodevelopmental disorders (NDDs), norm-referenced assessments, language, standardized assessment

## Abstract

Standardized, norm-referenced language assessment tools are used for a variety of purposes, including in education, clinical practice, and research. Unfortunately, norm-referenced language assessment tools can demonstrate floor effects (i.e., a large percentage of individuals scoring at or near the lowest limit of the assessment tool) when used with some groups with neurodevelopmental disorders (NDDs), such as individuals with intellectual disability and neurogenetic syndromes. Without variability at the lower end of these assessment tools, professionals cannot accurately measure language strengths and difficulties within or across individuals. This lack of variability may be tied to poor representation of individuals with NDDs in normative samples. Therefore, the purpose of this study was to identify and examine common standardized, norm-referenced language assessment tools to report the representation of individuals with NDDs in normative samples and the range of standard/index scores provided. A systematic search identified 57 assessment tools that met inclusion criteria. Coding of the assessment manuals identified that most assessment tools included a “disability” or “exceptionality” group in their normative sample. However, the total number of individuals in these groups and the number of individuals with specific NDDs was small. Further, the characteristics of these groups (e.g., demographic information; disability type) were often poorly defined. The floor standard/index scores of most assessment tools were in the 40s or 50s. Only four assessment tools provided a standard score lower than 40. Findings of this study can assist clinicians, educators, and researchers in their selections of norm-referenced assessment tools when working with individuals with NDDs.

## Introduction

Because the development of language is critical to meeting the demands of everyday life, accurate assessment of language is critical for diagnosing primary language disorders, identifying secondary language difficulties across other neurodevelopmental disorders (NDDs), and ultimately developing effective, targeted intervention and treatment plans that include monitoring progress over time. However, most commonly used assessment tools were not developed specifically for individuals with NDDs, and professionals who work with this population are often left without much guidance as to which tools to select. Therefore, the purpose of this study is to identify and examine common standardized, norm-referenced assessment tools of language to report the representation of individuals with NDDs in normative samples and the range of standard/index scores provided. This information can assist clinicians, educators, and researchers in their selections of norm-referenced assessment tools when working with individuals with NDDs.

Neurodevelopmental disorders are common in the United States, with birth cohort data (*n* > 3.3 million children) reporting that by 8 years of age, 23.9% of publicly insured children and 11% of privately insured children had a diagnosis of one or more NDDs (Straub et al., 2022). NDDs include a range of conditions resulting from either a genetic or multifactorial etiology (i.e., a combination of genetic and environmental factors) that occur during the developmental period and that are characterized by delays in cognition, communication, behavior, and/or motor skills (American Psychiatric Association [APA], 2013; Van Herwegen et al., 2015; World Health Organization [WHO], 2019). These conditions impact personal, social, academic, and/or occupational functioning (American Psychiatric Association [APA], 2013; Van Herwegen et al., 2015; World Health Organization [WHO], 2019). Specific NDDs include intellectual disability, communication disorders, autism, attention-deficit/hyperactivity disorder (ADHD), neurodevelopmental motor disorders (e.g., developmental coordination disorder, stereotypic movement disorder, and tic disorders), specific learning disorders, and some neurogenetic syndromes (e.g., Down syndrome, fragile X syndrome, and Williams syndrome). Different NDDs often co-occur; for example, individuals with autism may also have an intellectual disability or ADHD, and individuals with Down syndrome typically also have an intellectual disability (American Psychiatric Association [APA], 2013; Van Herwegen et al., 2015).

Many individuals with NDDs experience difficulties with language, though the exact pattern of these difficulties varies across diagnoses and individuals (e.g., Luyster et al., 2011). Some individuals have an NDD in which the primary diagnosis is specific to language. For example, developmental language disorder (under the umbrella of communication disorders) is linked to difficulties with pragmatics and structural aspects of language (e.g., Reed, 2018). Other individuals may have a different primary NDD but still also have language difficulties. For example, ADHD is often associated with secondary language difficulties in pragmatics (e.g., Geurts and Embrechts, 2008; Hawkins et al., 2016; Helland et al., 2016). Individuals with intellectual disability and neurogenetic syndromes also experience a range of difficulties in spoken language (Abbeduto et al., 2016; McDuffie et al., 2017), but the exact patterns of strength and difficulty often vary across different etiologies. For example, individuals with Down syndrome typically have relative strengths in vocabulary but more significant difficulties in grammar and syntax, whereas individuals with Williams syndrome tend to have relative strengths in concrete vocabulary but difficulties with relational vocabulary and pragmatics (Abbeduto et al., 2019).

One of the most common ways to measure language abilities is *via* standardized, norm-referenced language assessment tools. Norm-referenced assessment tools refer to those that have been tested (i.e., “normed”) on a large group of individuals meant to represent the age and demographic makeup of those for whom the test is intended to be used (Peña et al., 2006). When the assessment is administered in a standardized way, as outlined in the administration manual, an individual’s performance can then be compared to that of the normative sample to see how the individual compares to peers of a similar age and demographic makeup. However, the exact makeup of the normative sample can influence the scores of a norm-referenced assessment tool and its outcomes for the individual who is assessed (Peña et al., 2006; Spaulding et al., 2006). Thus, which norm-referenced language assessment tool a professional should use depends on the purpose of the assessment and the individual being assessed.

Two primary purposes of language assessment are to (1) diagnose language disorders and (2) describe language profiles. When the primary purpose of a language assessment is for diagnosis, a professional may want to select a norm-referenced assessment tool that did not include individuals with disabilities in the normative sample. Individuals with a primary language disorder may exhibit subtle, yet meaningful, language delays in which scores fall close to the diagnostic cut-off. In these cases, if individuals with disorders were included in the normative sample of the assessment tool being used, the normative group mean would be lower, with an increased standard deviation, resulting in decreased classification accuracy for identifying language impairment (i.e., a missed diagnosis), as demonstrated in a simulation study by Peña et al. (2006). On the other hand, for individuals with NDDs whose primary diagnosis is something other than a communication disorder (e.g., intellectual disability), the purpose of a language assessment is not typically for diagnosis but rather to describe their language profile and/or to identify their areas of strength or difficulty. This information can be used to guide intervention and academic planning. In these instances, it is important that norm-referenced assessment tools are not only reliable and valid for use in this population but that they also capture a wide range of skill levels, including at the lower-performing end where individuals with intellectual disability often fall.

Unfortunately, many standardized, norm-referenced assessment tools are not normed beyond three or four standard deviations below the normative mean, causing many participants with NDDs, such as individuals with intellectual disability, to score at the floor on standard/index scores (e.g., cf. Spaulding et al., 2006; Kasari et al., 2013; DiStefano et al., 2020). Floor effects occur when a large percentage of individuals have standard scores at or near the lowest limit of an assessment tool because its measurement range does not extend low enough to capture low levels of skills/performance (Hessling et al., 2004; McBee, 2010; Zhu and Gonzalez, 2017). Floor effects limit variability or separation in standard scores at the lower end of the assessment tool, and information regarding true differences across individuals is lost. These compressed scores, in turn, prevent researchers, clinicians, and educators from accurately capturing language strengths and difficulties within or across individuals and from tracking if individuals make clinically meaningful change/gains over time (Hessl et al., 2009; Sansone et al., 2014; Esbensen et al., 2017).

This issue of compressed scores is reflected in recent calls for the development of appropriate outcome measures for individuals with intellectual disability and neurogenetic syndromes (Esbensen et al., 2017; Hendrix et al., 2020). Floor effects have even been linked to recent failed pharmacological clinical trials for individuals with neurogenetic syndromes (Berry-Kravis et al., 2013; Budimirovic et al., 2017; Esbensen et al., 2017; see Abbeduto et al., 2020 for an overview; Baumer et al., 2022). Thus, many researchers, clinicians, and educators working with individuals with NDDs are pushing to develop more sensitive measures for use with these populations. Although there has been research addressing floor effects in cognitive/IQ tests (Hessl et al., 2009; Sansone et al., 2014), this line of research has not yet been extended to norm-referenced language assessment tools. At the same time, there is, and will continue to be, a need to use norm-referenced language assessment tools with this population, especially in clinical practice. Therefore, professionals who are assessing individuals who have an NDD that is *not* a primary language disorder and who are likely to score at the lower level of the assessment (e.g., intellectual disability) should select a norm-referenced assessment tool that has a low floor, to improve their ability to identify areas of strength and difficulty.

Given the variability in language skills across and within individuals with NDDs, and the various purposes of norm-referenced language assessment tools, researchers, educators, and clinicians need to be able to make informed decisions to select assessment tools that best meet their needs. Some may need norm-referenced assessment tools that did not include individuals with NDDs in their normative samples for better classification/diagnostic accuracy. Others may need norm-referenced assessment tools that have included individuals with NDDs in their normative samples, or at least that demonstrate variability at lower-performing ends of the assessment tool. Unfortunately, information on normative samples and psychometric properties is often not easily accessible before purchase, making it difficult to identify if a specific norm-referenced assessment tool meets one’s needs. Therefore, the purpose of this study was to:

1) Identify common standardized, norm-referenced assessment tools of language.
2) Report the representation of individuals with NDDs in their normative samples and the range of standard/index scores available.

## Materials and Methods

### Inclusion Criteria

To be included in our review, language assessment tools had to be a direct measure of oral language (e.g., the assessment tool could focus on any aspect of oral language, including phonology, but could not focus exclusively on articulation/speech or mostly on academics), have been published in the last 20 years (i.e., in or after 2002), and have been normed in the United States for individuals 22 years or younger (i.e., covers the developmental period; Schalock et al., 2021). In addition, the measure had to be published in English and commercially available for purchase by a main publishing house in the United States. Five main publishing houses in the United States were identified for review: Brookes Publishing, PARInc, Pearson, ProEd, and WPS. Screeners and caregiver-, teacher-, or self-report measures were not included.

### Procedures

#### Identification of Assessment Tools

Each of the five publishing houses’ websites was reviewed by two independent research assistants. The research assistants reviewed all assessment tools listed or tagged on the website as “speech and language” (or similar). Using the search function, they also searched for each of the following terms: “language,” “grammar,” “syntax,” “morphology,” “vocabulary,” “phonology,” “pragmatics,” “listening comprehension,” and “auditory processing.” Research assistants excluded any assessment tools that clearly did not meet the inclusion criteria but defaulted to including any language assessment tools that were unclear as to whether or not they met the study’s inclusion criteria. The first three authors made the final decisions on which assessment tools to include in situations of discrepancies across reviewers or when all reviewers were unsure. This process resulted in the identification of 55 assessment tools.

The assessment tool list was then reviewed by one university speech-language clinic director and one speech-language pathology clinical assistant professor with expertise in school-age language disorders. The clinicians were asked to review the list of assessment tools to determine if any language assessment tools were missed in the review. This process resulted in the inclusion of two additional assessment tools for a total of 57 assessment tools.

#### Coding of Assessment Tools

Following the identification of assessment tools, each assessment tool’s administration or technical manual was independently reviewed and coded by two research assistants for the variables listed below. Discrepancies were identified and resolved by the first and fourth author, with assistance from research assistants, by consulting the assessment tool manual.

### Variables

#### Full Normative Samples

The full normative sample of each assessment tool was coded for the total sample size and demographic information, including sex/gender, race/ethnicity, and geographic region. Each assessment tool was also coded for the chronological ages it was normed for and if the socioeconomic status of its normative sample was considered/reported.

#### Standard/Index Scores

We also documented the minimum and maximum standard/index scores provided by each assessment tool.

#### Inclusion of Individuals With Disabilities and Specific Neurodevelopmental Disorders in the Normative Sample

Many assessment tools included individuals with “disabilities” or “exceptionalities” in their normative samples without clearly differentiating disability type. For this reason, each assessment tool was coded for the total number of individuals with disabilities included in the normative sample. When possible, this information was also reported by disability type, including specific NDDs (e.g., number with intellectual disability, autism spectrum disorder, ADHD, learning disability). Demographic information was also coded for disability groups.

## Results

### Full Normative Samples

Demographic information on the full normative samples is reported in Table 1. A majority (*n* = 45/57) of assessment tools had normative sample sizes of over 1,000 individuals with relatively equal numbers of males and females. These samples included individuals from all regions of the United States, though five assessment manuals did not specify where their participants were from, and one did not have participants representing all regions of the United States. Sample diversity (defined in terms of race and ethnicity) was reported for all but one assessment tool [i.e., the Bilingual English-Spanish Assessment, BESA (Peña et al., 2018)] and varied across assessment tools. Most assessment tools (*n* = 49/57) considered some aspect of socioeconomic status (e.g., maternal education, income, and/or percentage receiving free or reduced lunch).

**TABLE 1:** Normative samples in norm-referenced language assessments.

Assessment	References	Ages normed	Range of standard/index scores	Sample size (*n*)	Sex/gender (%)	Race/ethnicity (%)^a^	Region (%)	SES considered
A Language Processing Skills Assessment, Fourth Edition (TAPS-4)	Martin et al., 2018	5:0–21:11	55–145	2023	Females (55), Males (45)	Race White/Caucasian (80), Black/African American (9), Asian American (4), American Indian/Alaska Native (1), Native Hawaiian/Pacific Islander (0.3), Two or more Ethnicities (6)	Northeast (15), Midwest (26), South (34), West (24)	Yes
						Ethnicity Hispanic (20) Non-Hispanic (80) Not reported (0.1)		

Arizona Articulation and Phonology Scale, Fourth Revision (Arizona-4)	Fudala and Stegall, 2017	1:6–21:11	≤ 50– ≥ 125	3192	Females (52), Males (48)	White (56), Black/African American (17), Asian (2), American Indian/Alaska Native (0.4), Native Hawaiian/Pacific Islander (0.3), Other (4), Hispanic Origin (20)	Northeast (13), Midwest (25), South (42), West (21)	Yes

Auditory Processing Abilities Test (APAT)	Ross-Swain and Long, 2004	5:0–12:11	55–145	1087	Females (51), Males (49)	White (72), Black (12), Asian (1), Other (3), Hispanic (12)	Northeast (17), Midwest (19), South (41), West (24)	No

Bankson Expressive Language Test - Third Edition (BELT-3)	Bankson et al., 2018	3:0–6:11	49–156	684	Females (50), Males (50)	Race White (72), Black/African American (15), Asian/Pacific Islander (5), American Indian/Eskimo/Aleut (1), Two or More (7)	Northeast (18), Midwest (22), South (37), West (23)	Yes
						Ethnicity Hispanic (24), Non-Hispanic (76)		

Bankson-Bernthal Test of Phonology, 2nd edition (BBTOP-2)	Bankson and Bernthal, 2020	3:0–9:11	40–128	770	Females (50), Males (50)	Race White (73), Black/African American (16), Asian/Native Hawaiian/Other Pacific Islander (3), American Indian/Alaska Native (< 1), Two or more (7)	Northeast (16), Midwest (23), South (37), West (24)	Yes
						Ethnicity Hispanic (22) Non-Hispanic (78)		

Bilingual English-Spanish Assessment (BESA)	Peña et al., 2018	4:0–6:11	< 55– > 145	756	Females (47), Males (42), Not reported (11)	Not specified	Northeast (29), South (47), West (24)	Yes

Clinical Assessment of Articulation and Phonology, Second Edition (CAAP-2)	Secord and Donohue, 2014	2:6–11:11	55–124	1486	Females (51), Males (49)	Race White (81) African American (13) Other (6)	Northeast (17), Midwest (21), South (38), West (24)	Yes
						Ethnicity Non-Hispanic (84) Hispanic (16)		

Clinical Assessment of Pragmatics (CAPs)	Lavi, 2019	7:0–18:11	64–136	914	Females (50), Males (50)	Race White (77), Black/African American (11), Asian (4), Other (7)	Northeast (19), Midwest (24), South (30), West (27)	Yes
						Ethnicity Hispanic (14)		

Clinical Evaluation of Language Fundamentals, Fifth Edition (CELF-5)	Wiig et al., 2013	5:0–21:11	40–160	2380	Females (50), Males (50)	White (57), African American (14), Asian (4), Other (6), Hispanic (20)	Northeast (15), Midwest (24), South (37), West (24)	Yes

Clinical Evaluation of Language Fundamentals Preschool, Third Edition (CELF-P3)	Wiig et al., 2020	3:0–6:11	40–160	700	Females (50), Males (50)	White (56) African American (13) Asian (2) Other (7) Hispanic (23)	Northeast (16), Midwest (23), South (44), West (18)	Yes

Communication and Symbolic Behavior Scales, Developmental Profile, First Normed Edition (CSBS), behavior sample	Wetherby and Prizant, 2002	1:0–2:0 Can be used up to 6:0 if developmental level is younger than 24 months	65–135	337	Females (49), Males (51)	Race White (87), Black (9), Asian (3), Other (0.9)	Not specified	Yes
						Ethnicity Hispanic (6), Non-Hispanic (94)		

Comprehensive Assessment of Spoken Language, Second Edition (CASL-2)	Carrow-Woolfolk, 2017	3:0–21:11	40–160	2394	Females (51), Males (49)	White (57), Black/African American (14), Asian (3), American Indian/Alaska Native (0.4), Native Hawaiian/Pacific Islander (0.3), Other (3), Hispanic Origin (22)	Northeast (23), Midwest (16), South (37), West (25)	Yes

Comprehensive Receptive and Expressive Vocabulary Test, Third Edition (CREVT-3)	Wallace and Hammill, 2013	5:0–89:11	45–155	1535	Females (49), Males (51)	Race White (80), Black/African American (14), Asian/Pacific Islander (4), American Indian/Eskimo/Aleut (< 1), Two or more (2)	Northeast (18), Midwest (20), South (37), West (25)	Yes
						Ethnicity Hispanic (15), Non-Hispanic (85)		

Comprehensive Test of Phonological Processing, Second Edition (CTOPP-2)	Wagner et al., 2013	4:0–24:11	43–165	1900	Females (51), Males (49)	Ethnicity White (76), Black/African American (14), Asian/Pacific Islander (2), Two or More (4), Other (4)	Northeast (18), Midwest (23), South (35), West (24)	Yes
						Hispanic Hispanic (16), Non-Hispanic (84)		

Diagnostic Evaluation of Articulation and Phonology (DEAP)	Dodd et al., 2006	3:0–8:11	55–145	650	Females (50), Males (50)	White (60), African American (15), Asian (4), Other (3), Hispanic (20)	Northeast (17), Midwest (21), South (37), West (26)	Yes

Emerging Literacy & Language Assessment (ELLA)	Wiig and Secord, 2006	4:6–9:11	< 55–163	1267	Females (53), Males (47)	White (60), African American (20), Other (9), Hispanic (11), Not specified (0.2)	Northeast (28), Midwest (20), South (35), West (16)	Yes

Expressive Language Test - Second Edition: Normative Update (ELT-2:NU)	Bowers et al., 2018b	5:0–11:11	46–149	1007	Females (49), Males (51)	Race White (77), Black/African American (17), Asian/Pacific Islander (6)	Northeast (18), Midwest (23), South (38), West (22)	No
						Ethnicity Hispanic (24), Non-Hispanic (76)		

Expressive One-Word Picture Vocabulary Test, Fourth Edition – English (EOWPVT-4)	Martin and Brownell, 2011a	2:0–70:0 +	< 55– > 145	2394	Females (56), Males (44)	Caucasian (63), African American (13), Asian American (3), Native American (1), Other (0.3), Hispanic (18), Not reported (1)	Northeast (24), Midwest (18), South (32), West (27)	Yes

Expressive One-Word Picture Vocabulary Test, Fourth Edition – Spanish - Bilingual Edition (EOWPVT-4:SBE)	Martin, 2013	2:0–70:0 +	< 55– > 145	1260	Females (55), Males (45)	Ethnicity Hispanic (94) White/Caucasian (4), African American (0.6), Native American (0.5), Asian American (0.1), Other (1), Not Reported (0.2)	Northeast (8), Midwest (15), South (36), West (42)	Yes
						Hispanic Origin Mexico (62), Puerto Rico (10), South America (6), Central America (6), Cuba (5), Dominican Republic (3), Haiti (0.6)		

Expressive Vocabulary Test, Third Edition (EVT-3)	Williams, 2018	2:6–90:0 +	40–160	2720	Not specified	White (62), African American (14), Asian (3), Other (5), Hispanic (17)	Northeast (13), Midwest (21), South (49), West (17)	Yes

Hodson Assessment of Phonological Patterns, Third Edition (HAPP-3)	Hodson, 2004	3:0–7:11	< 55–114	886	Females (49), Males (51)	Race White (76), Black (16), Other (8)	Northeast (19), Midwest (28), South (35), West (18)	Yes
						Ethnicity Hispanic (10), Non-Hispanic (90)		

Khan-Lewis Phonological Assessment, Third Edition (KLPA-3) & Goldman-Fristoe Test of Articulation, Third Edition (GFTA-3)	Khan and Lewis, 2015; Goldman and Fristoe, 2015	2:0–21:11	40–140	1500	Females (50), Males (50)	White (57), African American (11), Asian (2), Other (7), Hispanic (22)	Northeast (13), Midwest (24), South (41), West (23)	Yes

Language Processing Test 3: Elementary (LPT 3)	Richard and Hanner, 2005	5:0–11:11	< 40 - 150	1313	Females (50), Males (50)	Caucasian (61), African American (17), Asian American and Others (4), Hispanic (18)	Not specified	Yes

Listening Comprehension Test - Adolescent: Normative Update (LCT-A: NU)	Bowers et al., 2018a	12:0–17:11	48–136	1008	Females (50), Males (50)	Race White (77), Black/African American (16), Asian/Pacific Islander (4), Other (3)	Northeast (17), Midwest (24), South (36), West (23)	No
						Ethnicity Hispanic (21), Non-Hispanic (79)		

Listening Comprehension Test, Second Edition (LCT-2)	Bowers et al., 2006	6:0–11:11	< 62–159	1504	Females (50), Males (50)	Caucasian (63), African American (15), Hispanic (17), Asian American and Others (5)	Not specified	Yes

Montgomery Assessment of Vocabulary Acquisition (MAVA) - Expressive Vocab Test	Montgomery, 2008a	3:0–12:11	< 55– > 145	1248	Females (49), Males (52)	White (63), African American (16), Other (6), Hispanic (15)	Northeast (18), Midwest (25), South (36), West (21)	Yes

Montgomery Assessment of Vocabulary Acquisition (MAVA) - Receptive Vocab Test	Montgomery, 2008b	3:0–12:11	< 55– > 145	1373	Females (48), Males (52)	White (62), African American (17), Other (5), Hispanic (16)	Northeast (16), Midwest (23), South (40), West (20)	Yes

Oral and Written Language Scales, Second Edition (OWLS-II)	Carrow-Woolfolk, 2011	3:0–21:11	40–160	2123	Females (51), Males (49)	White (55), Black/African American (18), Asian (2), Native American (0.5), Native Hawaiian/Pacific Islander (0.4), Two or more races (4), Other (1), Hispanic origin (any race) (19)	Northeast (22), Midwest (22), South (37), West (19)	Yes

Oral Passage Understanding Scale (OPUS)	Carrow-Woolfolk and Klein, 2017	5:0–21:11	40–160	1517	Females (51), Males (49)	White (55), Black/African American (15), Asian (3), American Indian/Alaska Native (0.6) Native Hawaiian/Pacific Islander (0.4) Two or More/Other (4) Hispanic (22)	Northeast (20), Midwest (20), South (35), West (24)	Yes

Peabody Picture Vocabulary Test, Fifth Edition (PPVT-5)	Dunn, 2019	2:6–90:11 +	40–160	2720	Not specified	White (62), African American (14), Asian (3), Other (5), Hispanic (17)	Northeast (13), Midwest (21), South (49), West (17)	Yes

Phonological and Print Awareness Scale (PPA Scale)	Williams, 2014	3:6–8:11	< 50– > 130	1104	Females (51), Males (49)	Race White (76), Black/African American (16), Asian (4), Native American (0.4), Native Hawaiian/Pacific Islander (0.3), Other (3)	Northeast (28), Midwest (16), South (35), West (21)	Yes
						Ethnicity Hispanic (25), Non-Hispanic (76)		

Phonological Awareness Test, Second Edition: Normative Update (PAT-2: NU)	Robertson and Salter, 2018	5:0–9:11	39–123	1193	Females (49), Males (51)	Race White (77), Black/African American (17), Asian/Pacific Islander (3), Other (2)	Northeast (19), Midwest (24), South (37), West (21)	No
						Ethnicity Hispanic (23), Non-Hispanic (77)		

Preschool Language Assessment Instrument - Second Edition (PLAI-2)	Blank et al., 2003	3:0–5:11	49–160	463	Females (49), Males (51)	Race White (79), Black (15), Other (6)	Northeast (18), Midwest (24), South (35), West (23)	Yes
						Ethnicity African American (15), Hispanic American (13), Asian American (3), Native American (2), Other (67)		

Preschool Language Scales, Fifth Edition (PLS-5)	Zimmerman et al., 2011	birth–7:11	50 –150	1400	Females (50), Males (50)	White (54), African American (14), Asian (4), Hispanic (24), Other (4)	Northeast (20), Midwest (20), South (36), West (24)	Yes

Receptive One-Word Picture Vocabulary Test, Fourth Edition (ROWPVT-4)	Martin and Brownell, 2011b	2:0–80 +	< 55– > 145	2394	Females (56), Males (44)	Caucasian (63), African American (13), Asian American (3), Native American (1), Other (0.3), Hispanic (18), Not reported (1)	Northeast (24), Midwest (18), South (32), West (27)	Yes

Receptive, Expressive and Social Communication Assessment - Elementary (RESCA-E)	Hamaguchi and Ross-Swain, 2015	5:0–12:11	55– > 145	825	Females (50), Males (50) Not Reported (0.8)	Ethnicity White/Caucasian (77), Black/African American (13), Asian American (4), Native Hawaiian/Pacific Islander (0.4), American Indian/Alaska Native (0.1), Two or More (5), Not reported (0.2)	Northeast (18), Midwest (24), South (32), West (27)	Yes
						Hispanic Origin Hispanic (27), Non-Hispanic (73), Not reported (0.1)		

Social Language Development Test – Adolescent: Normative Update (SLDT-A: NU)	Bowers et al., 2017a	12:0–17:11	45–160	868	Females (50), Males (50)	Race White (77), Black/African American (18), Asian/Pacific Islander (4), Other (< 1)	Northeast (17), Midwest (22), South (38), West (23)	No
						Ethnicity Hispanic (21), Non-Hispanic (79)		

Social Language Development Test-Elementary: Normative Update (SLDT-E: NU)	Bowers et al., 2017b	6:0–11:11	47–160	1002	Females (49), Males (51)	Race White (78), Black/African American (15), Asian/Pacific Islander (5), Other (2)	Northeast (17), Midwest (23), South (38), West (22)	No
						Ethnicity Hispanic (19), Non-Hispanic (81)		

Structured Photographic Expressive Language Test, Third Edition (SPELT-3)	Dawson et al., 2003	4:0–9:11	< 40–142	1580	Females (48), Males (52)	Caucasian (66), African American (16), Hispanic (11), Other (7)	Northeast (19), Midwest (39), South (23), West (19)	Yes

The WORD Test, Third Edition: Elementary (WORD-3)	Bowers et al., 2014	6:0–11:11	–9–143	1302	Females (49), Males (51)	Caucasian (66), African American (13), Hispanic (17), Asian American and others (4)	Not specified	Yes

Test for Auditory Comprehension of Language, Fourth Edition (TACL-4)	Carrow-Woolfolk, 2014	3:0–12:11	45–160	1142	Females (49), Males (51)	Race White (78), Black/African American (14), Asian/Pacific Islander (3), American Indian/Eskimo/Aleut (1), Two or More (4)	Northeast (17), Midwest (22), South (36), West (25)	Yes
						Ethnicity Hispanic (20), Non-Hispanic (80)		

Test of Adolescent & Adult Language, Fourth Edition (TOAL-4)	Hammill et al., 2007	12:0–24:11	34–168	1671	Females (51), Males (49)	Ethnicity White (81), Black/African American (11), Asian/Pacific Islander (3), American Indian/Eskimo (2), Two or More (2), Other (1)	Northeast (21), Midwest (22), South (35), West (22)	Yes
						Hispanic Status Hispanic (12), Non-Hispanic (88)		

Test of Early Communication and Emerging Language (TECEL)	Huer and Miller, 2011	2 weeks–2:0	25–160	558	Females (50), Males (50)	Ethnicity White (78), Black/African American (10), Asian/Pacific Islander (5), American Indian/Eskimo (1), Two or More (6), Other (1)	Northeast (15), Midwest (19), South (34), West (32)	Yes
						Hispanic Status Hispanic (16), Non-Hispanic (84)		

Test of Early Language Development, Fourth Edition (TELD-4)	Hresko et al., 2018	3:0–7:11	50–155	1074	Females (50), Males (50)	Race White (75), Black/African American (14), Asian/Pacific Islander (5), American Indian/Eskimo (1), Two or more (5),	Northeast (17), Midwest (22), South (37), West (24)	Yes
						Ethnicity Hispanic (23), Non-Hispanic (77)		

Test of Expressive Language (TEXL)	Carrow-Woolfolk and Allen, 2014	3:0–12:11	47–159	1205	Females (51), Males (49)	Race White (78), Black/African American (15), Asian/Pacific Islander (4), American Indian/Eskimo/Aleut (1), Two or More Races (2)	Northeast (16), Midwest (22), South (36), West (26)	Yes
						Ethnicity Hispanic (21), Non-Hispanic (79)		

Test of Integrated Language and Literacy Skills (TILLS)	Nelson et al., 2016	6:0–18:11	40–145	1262	Females (50), Males (50)	White (Non-Hispanic) (73), African American (10), Asian (5), Native American (1) Other (1), Hispanic (Any Race) (10)	Northeast (5), Midwest (50), South (16), West (29)	Yes

Test of Language Development – Intermediate: Fifth Edition (TOLD-I:5)	Hammill and Newcomer, 2019	8:0–17:11	40 –167	1012	Females (51), Males (49)	Race White (74), Black/African American (14), Asian/Pacific Islander (4), American Indian/Alaska Native (2), Two or More (6)	Northeast (17), Midwest (21), South (36), West (26)	Yes
						Ethnicity Hispanic (25), Non-Hispanic (75)		

Test of Language Development – Primary, Fifth Edition (TOLD-P:5)	Newcomer and Hammill, 2019	4:0–8:11	41–165	1007	Females (47), Males (53)	Race White (71), Black/African American (13), Asian/Pacific Islander (6), American Indian/Alaska Native (3), Two or More (7)	Northeast (16), Midwest (20), South (36), West (28)	Yes
						Ethnicity Hispanic (25), Non-Hispanic (75)		

Test of Narrative Language, Second Edition (TNL-2)	Gilliam and Pearson, 2017	4:0–15:11	50–155	1310	Females (50), Males (50)	Race White (78), Black/African American (14), Asian/Pacific Islander (5), American Indian/Eskimo/Aleut (< 1) Two or More (2)	Northeast (16), Midwest (21), South (38), West (25)	Yes
						Ethnicity Hispanic (22), Non-Hispanic (78)		

Test of Phonological Awareness, Second Edition Plus (TOPA-2 +)	Torgeson and Bryant, 2004	5:0–8:11	49 –143	2085	Females (48), Males (52)	Race White (71), Black (16), Other (14)	Northeast (20), Midwest (24), South (34), West (23)	Yes
						Ethnicity White/European American (65), Black/African American (16), Asian/Pacific Islander (5), Native American/Eskimo/Aleut (2), Hispanic (15)		

Test of Pragmatic Language, Second Edition (TOPL-2)	Phelps-Terasaki and Phelps-Gunn, 2007	6:0–18:11	55–139	1136	Females (51), Males (49)	Race White (79), Black/African American (13), Asian/Pacific Islander (4), Native American (1), Two or More (2), Other (1)	Northeast (19), Midwest (23), South (35), West (23)	Yes
						Ethnicity Hispanic (13), Non-Hispanic (87)		

Test of Preschool Vocabulary (TOPV)	Mathews and Miller, 2015	2:0–5:11	54–160	1190	Females (49), Males (51)	Race White (74), Black/African American (13), Asian/Pacific Islander (5) American Indian/Eskimo/Aleut (1), Two or More (7)	Northeast (16), Midwest (22), South (38), West (24)	Yes
						Ethnicity Hispanic (23), Non-Hispanic (77)		

Test of Semantic Reasoning (TOSR)	Lawrence and Seifert, 2016	7:0–17:11	<55– > 145	1117	Females (49), Males (51)	Race White/Caucasian (71), Black/African American (19), Asian American (3), American Indian/Alaska Native (0.7), Native Hawaiian/Pacific Islander (0.4), Two or More (5), Not reported (0.5)	Northeast (13), Midwest (20), South (38), West (29)	Yes
						Ethnicity Hispanic (21), Non-Hispanic (79)		

Test of Semantic Skills - Intermediate: Normative Update (TOSS-I:NU)	Huisingh et al., 2019	9:0–13:11	52–143	1234	Females (49), Males (51)	Race White (77), Black/African American (18), Asian American/Native Hawaiian/Other Pacific Islander (4), American Indian/Alaska Native (2), Other (< 1)	Northeast (16), Midwest (24), South (36), West (24)	No
						Ethnicity Hispanic (20), Non-Hispanic (80)		

Test of Semantic Skills - Primary (TOSS-P)	Huisingh et al., 2002	4:0–8:11	<45–179	1510	Females (51), Males (49)	Caucasian (62), African American (17), Asian American and Others (5), Hispanic-American (16)	Not specified	No

Test of Word Finding, Third Edition (TWF-3)	German, 2014	4:6–12:11	45–132	1283	Females (49), Males (51)	Race White (77), Black/African American (14), Asian/Pacific Islander (4), American Indian/Eskimo/Aleut (1), Two or More (4)	Northeast (18), Midwest (25), South (35), West (22)	Yes
						Ethnicity Hispanic (19), Non-Hispanic (81)		

Vocabulary Assessment Scales – Expressive & Receptive (VAS-E & VAS-R)	Gerhardstein Nader, 2013	2:6–95:0	50–150	2678	Females (50), Males (50)	Caucasian (63), African American (13), Other (5), Hispanic (19)	Northeast (17), Midwest (19), South (52), West (13)	Yes

*^a^Race and ethnicity categories for each assessment are reported as they were presented in each manual.*

### Standard/Index Scores

The floor standard/index score of most assessment tools was in the 40s (*n* = 27/57) or 50s (*n* = 23/57). Three assessment tools had floor scores in the 60s [i.e., Clinical Assessment of Pragmatics, CAPs (Lavi, 2019); Communication and Symbolic Behavior Scales, Normed Edition, CSBS (Wetherby and Prizant, 2002); Listening Comprehension Test, LCT-2 (Bowers et al., 2006)]. Only four measures from our list provide a standard score lower than 40. The Phonological Awareness Test, Second Edition: Normative Update (PAT-2:NU; Robertson and Salter, 2018) provides standard scores down to 39. The WORD Test 3 Elementary (WORD-3; Bowers et al., 2014) provides scores down to −9. The Test of Adolescent and Adult Language (TOAL-4; Hammill et al., 2007) provides scores down to 34, and the Test of Early Communication and Emerging Language (TECEL; Huer and Miller, 2011) provides standard scores down to 25.

### Inclusion of Individuals With Disabilities and Specific Neurodevelopmental Disorders in the Normative Sample

#### Number of Individuals With Disabilities and Neurodevelopmental Disorders in Normative Samples

Of the 57 assessment tools, 52 indicated that they included individuals with disabilities in the normative sample (numbers and percentages of individuals with disabilities and specific NDDs are reported in Table 2). However, five assessment tools did not include or report on any individuals with disabilities in their normative sample: the BESA (Peña et al., 2018), the Test of Integrated Language and Literacy Skills (TILLS; Nelson et al., 2016), the Test of Phonological Awareness, Second Edition Plus (TOPA-2+; Torgeson and Bryant, 2004), the Test of Semantic Skills Primary (TOSS-P; Huisingh et al., 2002), and the Vocabulary Assessment Scales – Expressive (VAS-E) and Receptive (VAS-R; Gerhardstein Nader, 2013). These assessment tools are therefore not included in Table 2.

**TABLE 2:** Individuals with disabilities in normative samples.

Assessment	Disability Total Sample Size (*n*)^a^	% Normative Sample^b^	Diagnosis (*n*)^c^	% Normative Sample by Diagnosis	Sample Description
A Language Processing Skills Assessment (TAPS-4)	179	9	Specific Learning Disability/Dyslexia	2	Disability *Note: “Any Disability” represents the total number of individuals in the sample reporting one or more disability status categories.*
		
			Attention-Deficit Hyperactivity Disorder	2	
		
			Auditory Processing Disorder	0.4	
		
			Specific Language Impairment	3	
		
			Cochlear Implant/Hearing Impairment	2	
		
			Any Disability	9	

Arizona Articulation and Phonology Scale, Fourth Revision (Arizona - 4)	Not specified	7	Speech/Language Impairment	3	Individuals with severe disabilities (e.g., intellectual disability, moderate to severe autism spectrum disorder) were excluded from the standardization sample, whereas those with mild disabilities were included as long as they spent most of their day in a general education (not gifted or special education) classroom… 7% of the standardization sample had a diagnosed disability (3% speech/language impairment and 4% other diagnoses, including learning disability, developmental disability, intellectual disability, hearing/vision impairment, autism spectrum disorder, emotional disturbance, or other physical/health impairment; these other diagnoses each occurred with a frequency of 1% or less).
		
			Learning Disability	<1	
		
			Developmental Disability	<1	
		
			Intellectual Disability	<1	
		
			Autism Spectrum Disorder	<1	
		
			Hearing/Vision Impairment	<1	
		
			Emotional Disturbance	<1	
		
			Other Physical/Health Impairment	<1	
		

Auditory Processing Abilities Test (APAT)	Not specified	9-16^d^	Learning Disability	2	Disability Status *Note: 84% of the sample listed as having “no disability.”*
		
			Speech-Language Disorder	3	
		
			Other	4	

Bankson Expressive Language Test - Third Edition (BELT-3)	Not specified	<8	Intellectual Disability	<1	Exceptionality Type
		
			Attention-Deficit/Hyperactivity Disorder	1	
		
			Developmental Delay	1	
		
			Speech-Language Impairment	3	
		
			Learning Disability	<1	
		
			Autism Spectrum Disorder	<1	

Bankson-Bernthal Test of Phonology, 2nd edition (BBTOP-2)	Not specified	<9	Speech-Language Impaired	3	Exceptionality Status
		
			Attention-Deficit/Hyperactivity Disorder	2	
		
			Learning Disabled	1	
		
			Developmentally Delayed	1	
		
			Intellectually Disabled	<1	
		
			Autism Spectrum Disorder	1	

Clainical Assessment of Articulation and Phonology, Second Edition (CAAP-2)	Not specified	7	Not specified	Not specified	Seven percent of the standardization subjects were receiving speech and language services.

Clinical Assessment of Pragmatics (CAPs)	137	15	Autism Spectrum Disorder	2	Clinical Groups
		
			Specific Language Impairment	3	
		
			Other	10	

Clinical Evaluation of Language Fundamentals, Fifth Edition (CELF-5)	Not specified	<26	Attention Deficit Disorders	5	Of the students in the standardization sample, 11% reported the following diagnoses: 5% attention deficit disorders (inattentive, hyperactive, and combined); 1% learning disability; 1% intellectual disability, pervasive developmental disorder, Down syndrome, or developmental delay; and less than 1% each emotional disturbance, cerebral palsy, color blindness, central auditory processing disorder, visual impairment, autistic spectrum disorder, or other diagnoses not specified. Approximately 3% of the sample was receiving occupational or physical therapy. Approximately 12% of the sample was diagnosed with a speech and/or language disorders; of those, 7.2% reported diagnoses of language disorder (including receptive/expressive language disorder or pragmatics impairment), 4.2% reported articulation or phonological disorder, and less than 1% reported fluency and voice disorder.
		
			Learning Disability	1	
		
			Intellectual Disability, Pervasive Developmental Disorder, Down syndrome, and Developmental Delay	1	
		
			Emotional Disturbance	<1	
		
			Cerebral Palsy	<1	
		
			Color blindness	<1	
		
			Central Auditory Processing Disorder	<1	
		
			Visual Impairment	<1	
		
			Autistic Spectrum Disorder	<1	
		
			Other Diagnosis Not Specified	<1	
		
			Language Disorder	7	
		
			Articulation or Phonological Disorder	4	
		
			Fluency and Voice Disorder	<1	
		
			Occupational or Physical Therapy	3	

Clinical Evaluation of Language Fundamentals Preschool, Third Edition (CELF-P3)	Not specified	7	Occupational or Physical Therapy	1	According to the inclusion and exclusion specifications for the normative sample, the children included did not meet the diagnosis criteria for a language impairment, a learning disorder in reading or writing, or a hearing impairment… To reflect the variability in learning needs that naturally occur in the general population, a limited number of children with special education placement were included in the normative sample. Approximately 8% of children in the sample were reported as receiving special services: less than 1% for gifted and talented, 1.3% for occupational or physical therapy, 2% early childhood or other services, and an overlapping 4% received services for both speech and language.
		
			Early Childhood and Other Services	2	
		
			Services in Both Speech and Language	4	

Communication and Symbolic Behavior Scales, Developmental Profile, First Normed Edition (CSBS)	Not specified	Not specified	Not specified	Not specified	Children with known developmental delays or who qualified for Part C early intervention services were excluded from the standardization sample… Because of the extent of the under-identification of children with developmental delays from birth-24 months of age, it is presumed that at least 10% of the standardization sample has developmental delays or disabilities, although children with severe disabilities are likely not included in this sample.

Comprehensive Assessment of Spoken Language, Second Edition (CASL-2)	Not specified	Not specified	Not specified	Not specified	Individuals with severe disabilities (e.g., intellectual disability, moderate to severe autism spectrum disorder) were excluded from the standardization sample, while those with mild disabilities were included as long as they spent most of their school day in a general education classroom (not gifted or special education), at a grade level appropriate to the child’s chronological age.

Comprehensive Receptive and Expressive Vocabulary Test, Third Edition (CREVT-3)	Not specified	25	Learning Disability	5	Exceptionality Status
		
			Articulation Disorder	5	
		
			Language Impaired	5	
		
			Attention-Deficit Disorder	4	
		
			Other	6	

Comprehensive Test of Phonological Processing, Second Edition (CTOPP-2)	Not specified	<7	Specific Learning Disabilities	1	Exceptionality Status
		
			Intellectual Disability	<1	
		
			Hearing Impairment	<1	
		
			Other Health Impairment	<1	
		
			Attention-Deficit Disorder	2	
		
			Other Disability	1	

Diagnostic Evaluation of Articulation and Phonology (DEAP)	52	8	Disability (subgroup percentages not reported)	8	The DEAP norms were developed on a sample that included 650 children; 94.3% had no speech or language disorder and 5.7% had diagnosed articulation, phonological, or oral motor disorders… Eight percent of children in the normative sample were reported by parents and examiners to be diagnosed with one or more of the following: receptive and/or expressive language disorder, articulation disorder, phonological disorder, oral motor disorder, Attention-deficit/hyperactivity disorder, cerebral palsy, developmental delay, fluency disorder, learning disability, orthopedic handicap, visual impairment, and other health impairment. Less than 1 percent were identified as gifted.

Emerging Literacy & Language Assessment (ELLA)	Not specified	Not specified	Not specified	Not specified	Some children with language disorders, learning disabilities, and some children receiving remediation services in reading (but not special education services) were included in the standardization sample.

Expressive Language Test - Second Edition: Normative Update (ELT-2:NU)	Not specified	14	Language Impairment	4	Exceptionality Status *Note: 86% of the sample listed as having “no disability.”*
		
			Other Disability	10	

Expressive One-Word Picture Vocabulary Test, Fourth Edition -English (EOWPVT-4)	298	12	Any Disability	12	Information regarding disability status is from the U.S. Department of Education (2000).

Expressive One-Word Picture Vocabulary Test, Fourth Edition - Spanish-Bilingual Edition (EOWPVT-4:SBE)	131	10	Any Disability	10	Not specified

Expressive Vocabulary Test, Third Edition (EVT-3)	Not specified	4	Attention-Deficit Disorders	0.8	According to the inclusion and exclusion specifications for the study, the individuals included in this sample did not meet the diagnosis criteria for language disorder, learning disorder, or hearing impairment. Review of each individual’s test results indicated expected performance for individuals without language and/or learning disability… To reflect the variability in learning needs that naturally occur in the general population, a limited number of individuals with special education placement were included in the normative sample. Of the sample, 0.8% were reported with an educational placement for gifted or talented. In addition, 3.7% of the individuals in the normative sample reported an educational diagnosis: approximately 0.8% attention deficit disorders (inattentive, hyperactive, and combined); 0.7% autism spectrum disorder; 0.2% developmental delay; 0.2% hearing impairment; 0.6% learning disability in reading and/or writing; 0.3% speech and/or language delay; and 0.9% speech and/or language disorder.
		
			Autism Spectrum Disorder	0.7	
		
			Developmental Delay	0.2	
		
			Hearing Impairment	0.2	
		
			Learning Disability in Reading and/or Writing	0.6	
		
			Speech and/or Language Delay	0.3	
		
			Speech and/or Language Disorder	0.9	

Hodson Assessment of Phonological Patterns, Third Edition (HAPP-3)	Not specified	3	Phonological Impairment	2	Disability Status *Note: 97% of the sample listed as having “no disability.”*
		
			Other Disability	1	

Khan-Lewis Phonological Assessment, Third Edition (KLPA-3) & Goldman-Fristoe Test of Articulation, Third Edition (GFTA-3)	Not specified	20	Speech and/or Language Disorder	8	20% were reported with the following diagnoses: approximately 8% speech and/or language disorder; 4% attention deficit disorders (inattentive, hyperactive, and combined); 3% learning disability; 2% intellectual disability, pervasive developmental disorder, Down syndrome, or developmental delay; and less than 1% each emotional disturbance, cerebral palsy, central auditory processing disorder, visual impairment, autistic spectrum disorder, or other diagnoses not specified.
		
			Attention Deficit Disorders	4	
		
			Learning Disability	3	
		
			Intellectual Disability, Pervasive Developmental Disorder, Down syndrome, or Developmental Delay	2	
		
			Emotional Disturbance	<1	
		
			Cerebral Palsy	<1	
		
			Central Auditory Processing Disorder	<1	
		
			Visual Impairment	<1	
		
			Autistic Spectrum Disorder	<1	
		
			Other diagnoses not specified	<1	

Language Processing Test 3: Elementary (LPT-3)	Not specified	Not specified	Not specified	Not specified	The sample included normal subjects and subjects with language-learning disorders. Subjects previously identified as having hearing impairment, mental disabilities, emotional disabilities, or limited English proficiency were excluded from the standardization sample.

Listening Comprehension Test - Adolescent: Normative Update (LCT-A: NU)	Not specified	12	Specific Language Impairment	4	Exceptionality status: *Note: Other/Special education consisted of students receiving special education services for a variety of conditions.*

			Other/Special Education	8	

Listening Comprehension Test, Second Edition (LCT-2)	Not specified	Not specified	Not specified	Not specified	Subjects from regular education; special education … were included in the study. In addition, subjects with IEPs for special services (e.g., articulation disorder, remedial reading) but who attend regular education classes were included. Subjects excluded from the study included those who were not able to use English proficiently at school, were non-verbal, had any degree of hearing loss, or who resided outside of the United States.

Montgomery Assessment of Vocabulary Acquisition (MAVA) - Expressive Vocab Test	Not specified	10	Not Specified	Not specified	Ten percent of this population included children in special education with known vocabulary deficits.

Montgomery Assessment of Vocabulary Acquisition (MAVA)- Receptive Vocab Test	Not specified	10	Not Specified	Not specified	Ten percent of this population included children in special education with known vocabulary deficits.

Oral and Written Language Scales, Second Edition (OWLS - II)	Not specified	Not specified	Not specified	Not specified	Individuals with diagnosed disabilities were included in the standardization sample as long as they spent most of their school day in a regular classroom. The percentage of such individuals matched what is expected in the population.

Oral Passage Understanding Scale (OPUS)	Not specified	Not specified	Not specified	Not specified	Individuals with severe disabilities (e.g., intellectual disability, moderate to severe autism) were excluded from the standardization sample, while those with mild disabilities were included as long as they spent most of their day in a general classroom.

Peabody Picture Vocabulary Test, Fifth Edition (PPVT-5)	Not specified	4	Attention Deficit Disorders	0.8	According to the inclusion and exclusion specifications for the study, the individuals included in this sample did not meet the diagnosis criteria for language disorder, learning disorder, or hearing impairment. Review of each individual’s test results indicated expected performance for individuals without language and/or learning disability… To reflect the variability in learning needs that naturally occur in the general population, a limited number of individuals with special education placement were included in the normative sample. Of the sample, 0.8% were reported with an educational placement for gifted or talented. In addition, 3.7% of the individuals in the normative sample reported an educational diagnosis: approximately 0.8% attention deficit disorders (inattentive, hyperactive, and combined); 0.7% autism spectrum disorder; 0.2% developmental delay; 0.2% hearing impairment; 0.6% learning disability in reading and/or writing; 0.3% speech and/or language delay; and 0.9% speech and/or language disorder.
		
			Autism Spectrum Disorder	0.7	
		
			Developmental Delay	0.2	
		
			Hearing Impairment	0.2	
		
			Learning Disability in reading and/or writing	0.6	
		
			Speech and/or Language Delay	0.3	
		
			Speech and/or Language Disorder	0.9	

Phonological and Print Awareness Scale (PPA Scale)	Not specified	Not specified	Not specified	Not specified	Children with mild disabilities were included in the standardization sample as long as they spent most of their school day in a regular classroom.

Phonological Awareness Test, Second Edition: Normative Update (PAT-2: NU)	Not specified	15	Language Impairment	3	Exceptionality Status *Note: 85% of the sample listed as having “no disability.”*
		
			Special Education	12	

Preschool Language Assessment Instrument - Second Edition (PLAI-2)	Not specified	11	Speech-Language Disorder	4	Disability Status *Note: 89% of the sample listed as having “no disability.”*
		
			Intellectual Disability	0	
		
			Other Handicap	7	

Preschool Language Scales - Fifth Edition (PLS-5)	90	6	Speech Language Disorder	4	Educational Classification/Diagnosis *Note: Other includes hearing impairments, other health impairments, visual impairments, multiple disabilities, deaf-blindness, and traumatic brain injury.*
		
			Intellectual Disability	0.1	
		
			Developmental Delay	1	
		
			Attention-Deficit/Hyperactivity Disorder	0.2	
		
			Orthopedic/Motor Impairment	0.1	
		
			Other	0.9	

Receptive One-Word Picture Vocabulary Test, Fourth Edition (ROWPVT-4)	298	12	Any Disability	12	Information regarding disability status is from the U.S. Department of Education (2000).

Receptive, Expressive and Social Communication Assessment - Elementary (RESCA-E)	126	15	Autism Spectrum Disorder	4	Disability *Note: “Any Disability” represents the total number of individuals in the sample reporting one or more disability status categories.*
		
			Attention Deficit/Hyperactivity Disorder	2	
		
			Developmental Disability	3	
		
			Emotional Disturbance	1	
		
			Intellectual Disability	0.7	
		
			Social Communication Disorder	1	
		
			Speech and Language Impairment	5	
		
			Learning Disability	4	
		
			Any Disability	15	

Social Language Development Test – Adolescent: Normative Update (SLDT-A: NU)	Not specified	20	Specific Language Impairment	6	Exceptionality Status *Note: Other/Special Education subgroup consisted of students receiving special education services for a variety of conditions.*
		
			Autism Spectrum Disorder	5	
		
			Other/Special Education	9	

Social Language Development Test-Elementary: Normative Update (SLDT-E: NU)	Not specified	17	Specific Language Impairment	5	Exceptionality Status *Note: Other/Special Education subgroup consisted of children receiving special education services for a variety of conditions.*
		
			Autism Spectrum Disorder	2	
		
			Other/Special Education	10	

Structured Photographic Expressive Language Test - Third Edition (SPELT-3)	Not specified	> 7	Not specified	Not Specified	Slightly more than 7% of the sample was identified as language impaired consistent with prevalence estimates of 7% in the population (Leonard, 1998) and 7.4% (Tomblin et al., 1997).

The WORD Test, Third Edition: Elementary (WORD-3)	Not specified	Not specified	Not specified	Not specified	In addition, subjects with Individualized Education Plans (IEPs) for special services (e.g., articulation disorder, remedial reading) but who attended regular education classes were included. Subjects excluded from this study included those who were not able to use English proficiently at school, were non-verbal, had any degree of hearing loss, or who resided outside of the United States.

Test for Auditory Comprehension of Language, Fourth Edition (TACL-4)	Not specified	18	Intellectual Disability	4	Exceptionality Type
		
			Deaf/Hard of Hearing	1	
		
			Language Impairment	4	
		
			Learning Disability	4	
		
			Attention-Deficit/Hyperactivity Disorder	3	
		
			Autism Spectrum Disorder	2	

Test of Adolescent & Adult Language, Fourth Edition (TOAL-4)	Not specified	15	Disabled	15	Exceptionality Status *Note: 85% of the sample listed as “not disabled.” The data on exceptionality status represent students being served under the Individuals with Disabilities Education Act and does not include those who have a language disorder, who have attention-deficit/hyperactivity disorder, or who are gifted and talented.*

Test of Early Communication and Emerging Language (TECEL)	47	8	Not specified	Not specified	The TECEL was normed on a sample of 558 persons (47 with disabilities).

Test of Early Language Development, Fourth Edition (TELD-4)	Not specified	13	Intellectual Disability	1	Exceptionality Status
		
			Developmental Disability	2	
		
			Speech/Language Impairment	6	
		
			Learning Disability	2	
		
			Attention-Deficit/Hyperactivity Disorder	1	
		
			Autism Spectrum Disorder	1	

Test of Expressive Language (TEXL)	Not specified	16	Intellectual Disability	2	Exceptionality Type
		
			Language Impairment	3	
		
			Articulation Disorder	3	
		
			Learning Disability	4	
		
			Attention-Deficit Hyperactivity Disorder	2	
		
			Autism Spectrum Disorder	1	
		
			Deaf/Hard of Hearing	1	

Test of Language Development – Intermediate: Fifth Edition (TOLD-I:5)	Not specified	<21	Intellectual Disability	1	Exceptionality Status
		
			Deaf/Hard of Hearing	<1	
		
			Attention-Deficit/Hyperactivity Disorder	4	
		
			Articulation Disorder	2	
		
			Asperger Syndrome/High-Functioning Autism	<1	
		
			Developmental Delay	<1	
		
			Emotional/Behavior Disorder	2	
		
			Specific Learning Disability	5	
		
			Language Impairment	2	
		
			Low-Functioning Autism	<1	
		
			Other Disability	<1	

Test of Language Development – Primary, Fifth Edition (TOLD-P:5)	Not specified	<20	Intellectual Disability	<1	Exceptionality Status
		
			Attention-Deficit/Hyperactivity Disorder	<1	
		
			Articulation Disorder	5	
		
			Asperger Syndrome/High-Functioning Autism	1	
		
			Developmental Delay	2	
		
			Behavior Disorder	1	
		
			Learning Disability	4	
		
			Language Impairment	3	
		
			Low-Functioning Autism	1	
		
			Other Disability	1	

Test of Narrative Language, Second Edition (TNL-2)	Not specified	<11	Specific Learning Disabilities	2	Exceptionality Status
		
			Intellectual Disability	3	
		
			Deaf/Hard of Hearing	<1	
		
			Other Health Impairments	<1	
		
			Attention-Deficit/Hyperactivity Disorder	2	
		
			Physically Impaired	1	
		
			Other Disability	1	

Test of Pragmatic Language, Second Edition (TOPL-2)	Not specified	23	Behavioral Disorder	<1	Disability/Exceptionality Status *Note: 77% of the sample listed as having “no exceptionality/disability.”*
		
			Developmental Delay	1	
		
			Asperger’s Syndrome	1	
		
			Articulation Disorder	2	
		
			Learning Disability	5	
		
			Attention-Deficit/Hyperactivity Disorder	2	
		
			Intellectual Disability	<1	
		
			Autism	<1	
		
			Emotional Disturbance	3	
		
			Physical Impairment	<1	
		
			Speech-Language Impairments	2	
		
			Deaf/Hard of Hearing	<1	
		
			Blind/Visual Impairments	<1	
		
			Traumatic Brain Injury	<1	

Test of Preschool Vocabulary (TOPV)	Not specified	15	Intellectual Disability	1	Exceptionality Status *Note: 85% of the sample listed as having “no exceptionality.”*
		
			Deaf/Hard of Hearing	1	
		
			Developmental Delay	6	
		
			Emotional Disturbance	<1	
		
			Behavioral Disorder	<1	
		
			Language Impairment	7	
		
			Autism Spectrum Disorder	2	

Test of Semantic Reasoning (TOSR)	114	10	Specific Language Impairment	3	Disability *Note: “Any Disability” is defined as total number of individuals in the sample reporting one or more disability status categories.*
		
			Learning Disability	3	
		
			Autism	2	
		
			Attention-Deficit/Hyperactivity Disorder	4	
		
			Any Disability	10	

Test of Semantic Skills - Intermediate: Normative Update (TOSS-I:NU)	Not specified	9	Language Impairment	3	Exceptionality Status *Note: Other/Special Education subgroup consisted of children receiving special education services for a variety of conditions.*
		
			Other/Special Education	6	

Test of Word Finding, Third Edition (TWF-3)	Not specified	14	Attention-Deficit/Hyperactivity Disorder	3	Exceptionality Status *Note: 86% of the sample listed as having “no disability.”*
		
			Specific Learning Disability	2	

			Speech or Language Impairment	3	
		
			Word Finding Problem	3	
		
			Intellectual Disability	<1	
		
			Other Disability	5	

*Percentages were rounded to the nearest whole number unless they were less than 1.*

*^a^Sample size (n) is only reported here if the n was provided in the assessment manual.*

*^b^Some assessments did not report the overall sample size (n) or overall percentage of individuals with disabilities in their manual but instead reported the sample size (n) or percentages of individual disability groups (e.g., n’s with learning disability, autism). When this happened, the overall percentage reported in the table was estimated based on the available information.*

*^c^Individual disability groups are reported as presented and labeled in each assessment manual. One exception is that the term “mental retardation” was updated to “intellectual disability”.*

*^d^Under the category of “Disability Status”, the APAT manual reported that 84% of the normative sample had “no disability”, 1.7% had a “learning disability”, 3.2% had a “speech-language disability”, and 4.3% had an “other” disability. Even if there were no dual-diagnoses, these percentages do not account for 100% of the normative sample.*

Nine assessment tools indicated that they may have included some individuals with disabilities, or alternatively did not exclude all individuals with disabilities. However, they did not track and/or specify if/how many individuals with disabilities were included. To be as inclusive as possible, these assessment tools are reported in Table 2.

For the remaining 43 assessment tools that clearly included individuals with disabilities in their normative samples, the total number varied across assessment tools. However, in most cases, this was a low percentage of the normative sample (ranging from 3 to <26%). Only six assessment tools had normative samples in which 20% or more of the normative sample had a disability or an “exceptionality status”: Clinical Evaluation of Language Fundamentals, Fifth Edition (CELF-5; Wiig et al., 2013), Comprehensive Receptive and Expressive Vocabulary Test, Third Edition (CREVT-3; Wallace and Hammill, 2013), Khan-Lewis Phonological Assessment, Third Edition (KLPA-3; Khan and Lewis, 2015), Social Language Development Test – Adolescent: Normative Update (SLDT-A:NU; Bowers et al., 2017a), Test of Language Development – Intermediate: Fifth Edition (TOLD-I:5; Hammill and Newcomer, 2019), Test of Pragmatic Language, Second Edition (TOPL-2; Phelps-Terasaki and Phelps-Gunn, 2007). Another 21 assessment tools had normative samples in which 10–19% had a disability. Fifteen assessment tools had normative samples in which less than 10% had disabilities, and one assessment tool [i.e., the Auditory Processing Abilities Test, APAT (Ross-Swain and Long, 2004)] had between 9 and 16%, though the exact percentage was unclear. Further, the overall sample size (*n*) of individuals with disabilities was not reported in all assessment tools. When possible, we estimated the overall percentage of individuals with disabilities based on the available information (e.g., reported *n*’s of specific NDDs). This method does not account for dual-diagnoses, though, so the reported number may be smaller than estimated.

#### Descriptions and Demographic Information of Individuals With Disabilities and Neurodevelopmental Disorders in Normative Samples

The makeup (i.e., disability type and demographic information) of individuals with disabilities was often poorly defined for these 43 assessment tools. Ten assessment manuals did not specify what type(s) of disabilities were represented in their normative sample (i.e., the number of individuals with specific disabilities or NDDs such as intellectual disability or learning disabilities). Another 10 assessment tools only reported 2–3 disability groups (e.g., language impairment and “other/special education”). Further, no assessment tools reported race, ethnicity, or socioeconomic status information specifically for subgroups of individuals with disabilities in their normative samples.

## Discussion

The purpose of this study was to identify the number and characteristics of individuals with NDDs in commonly used and commercially available standardized, norm-referenced language assessment tools. Our findings indicate that many of these assessment tools, though not all, did include some individuals with “disabilities” in their normative sample. However, the number of individuals with specific types of disabilities or NDDs was often very low, and minimal demographic information was provided about groups with disabilities.

We identified 43 assessment tools that included individuals with disabilities in their normative samples. These “disability” groups typically included individuals with *any* disabilities, not just NDDs, and the groups were often not broken down by disability type. Therefore, the number of individuals with NDDs, specifically, in the normative samples was often unclear. There was high variability in the percentages of individuals with “disabilities” in the normative samples, ranging from 3% to <26%. These rates align with some available prevalence data on individuals with disabilities in the United States. For example, 2019 United States census data reveal that 4.3% of children under 18 have a disability (Young, 2021), and 2020–2021 United States special education data indicate that 15% of 3-to-21-year-olds receive services under the Individuals with Disabilities Education Act (National Center for Education Statistics, 2022). However, the percentage of children with NDDs reported from public or private insurance data is even higher [i.e., 23.9 and 11%, respectively (Straub et al., 2022)]. It would be helpful if our results could be easily interpreted within the available prevalence data, but similar to the reporting of disabilities within the assessment tools we reviewed, these data are also difficult to interpret and vary based on how disabilities are defined. This presents a barrier to the selection of standardized assessment tools for these populations.

Another barrier is the lack of demographic information (i.e., race, ethnicity, and socioeconomic status) provided about the individuals with disabilities or NDDs in the normative samples of the assessment tools. Without this information, the diversity of the individuals in these groups is unknown. It is possible, for example, that there were no Black individuals with autism included in some normative samples or no Hispanic individuals with intellectual disability. Thus, it is unknown if the individuals with disabilities who were included are representative of these groups as a whole, including across race, ethnicity, and socioeconomic status. Together with the lack of definition of “disability” provided by many of the assessment tools we reviewed, it is unclear if their normative samples are representative of the population of individuals with disabilities or NDDs in the United States.

### Considerations for the Selection of Norm-Referenced Language Assessment Tools

There are several scenarios in which clinicians, educators, and researchers must use standardized, norm-referenced language assessment tools with individuals who have, or who are suspected of having, NDDs. The information extracted from this study can be used by these professionals to guide the selection of such assessment tools and while interpreting their scores.

The decision about which language assessment tools are most suitable depends on the specific population of interest and the intended purpose of the assessment. Professionals using norm-referenced assessments tools to identify if an individual has a *primary* diagnosis of a communication disorder may want to choose an assessment tool that does not include individuals with “disabilities” in the normative sample because they are trying to determine if an NDD (e.g., a communication disorder) is present or absent. To make this determination, an individual’s score should be compared to a normative sample of peers who *do not* have an NDD. Relatedly, professionals using norm-referenced assessment tools to determine if clients qualify for services (e.g., special education services) may also wish to use assessment tools that do not include individuals with disabilities in their samples, because, as Peña et al. (2006) demonstrated, the presence of individuals with disabilities in the normative sample can lower the level of performance that falls within the average range. Consequently, it becomes less likely that an individual who has a disability will score below the average range and thus be eligible for services. This is particularly important when evaluating an individual with relatively mild delays. In our review, this included the BESA (Peña et al., 2018), the TILLS (Nelson et al., 2016), the TOPA-2+ (Torgeson and Bryant, 2004), the TOSS-P (Huisingh et al., 2002), and the VAS-E and VAS-R (Gerhardstein Nader, 2013).

In contrast, professionals working with individuals with NDDs may select a standardized assessment tool for the purpose of identifying areas of strength and need to support intervention and educational planning. This may be common when an individual has a *primary* diagnosis of an NDD other than a communication disorder, in which language is also affected (e.g., intellectual disability). In such cases, it is ideal to select an assessment tool that has been developed and normed with others who have a similar NDD and who are demographically similar to their client. This is especially important when working with clients who have more severe disabilities and who are at risk of performing at the floor level of an assessment tool. Thus, yet another important consideration is the range and floor level of the standard/index scores. Those with lower floors may allow for more separation between scores at the lower-performing end. This, in turn, allows for greater differentiation across specific skills or language domains. These assessment tools are also better options for professionals who are using norm-referenced assessment tools to monitor progress over time (e.g., clinical gains or intervention success). In our review, we identified that the floor score of some language assessment tools is in the 60s, while others include scores between 50 and 40, and only four had standard scores of 40 or lower. Those with floors below 40 were the PAT-2:NU (Robertson and Salter, 2018), the WORD-3 (Bowers et al., 2014), the TOAL-4 (Hammill et al., 2007), and the TECEL (Huer and Miller, 2011).

Similarly, researchers who are documenting patterns of strength and difficulty to inform the field about different NDD phenotypes should also consider selecting norm-referenced assessment tools that have included individuals with NDDs and that have a wide range of standard/index scores with lower floors. This allows for more nuance in understanding the variability among participant samples, especially at the lower-performing end. The ability to include participant samples with more diverse language profiles can lead to more precise phenotyping that can ultimately be applied to develop evidence-based language interventions. This could also improve the likelihood of intervention success because intervention studies and clinical trials often fail to demonstrate response to treatment due in part to poor outcome measures (see Esbensen et al., 2017; Abbeduto et al., 2020). If language assessment tools can better differentiate among different language profiles, it may be possible for researchers to specify who does and does not respond to certain interventions. When researchers select measures that *do not* include individuals with NDDs in the normative sample, the interpretation of skills and abilities is reduced to comparisons with neurotypical peers. Instead, if individuals with NDDs are compared to individuals with other NDDs (e.g., Down syndrome vs. intellectual disability), areas of unique strength and need can be identified and used in treatment planning.

### Future Directions and Recommendations for Holistic Language Assessment for Individuals With Neurodevelopmental Disorders

Several researchers have noted the limited utility of standardized, norm-referenced assessment tools for individuals with certain NDDs (e.g., intellectual disability and neurogenetic syndromes) and have started developing more sensitive measures for these populations (e.g., Berry-Kravis et al., 2013; Budimirovic et al., 2017; Esbensen et al., 2017; Abbeduto et al., 2020; Baumer et al., 2022). For example, Brady et al. (2012, 2018, 2020) developed the Communication Complexity Scale to assess communication skills in individuals who have intellectual disabilities and are minimally speaking, and Abbeduto et al. (2020) and Thurman et al. (2021) developed an expressive language sampling procedure for use with individuals with intellectual disability and neurogenetic syndromes. These measures capture more variability in language and communication skills in individuals with intellectual and developmental disabilities, with demonstrated evidence of their usefulness as outcome measures. Thus, professionals working with individuals with NDDs should consider these measures when tracking progress over time. These assessment tools can also be used by professionals working with neurotypical individuals; for example, Channell et al. (2018) documented that expressive language sampling in the context of narration showed age-related increases in syntactic complexity and lexical diversity from 4 years up until 18.5 years. As these language assessment tools continue to be tested and examined, professionals may have more options in which to assess clients with NDDs.

In addition to these language/communication sampling assessment tools, there will continue to be a need for norm-referenced language assessment tools for use with individuals with NDDs. Thus, in the future, test developers should not only consider including a more representative number of individuals with NDDs in their normative samples but also as part of the iterative test development and standardization processes. Test developers should also consider the possibility of including separate norms for individuals with NDDs and/or who perform at the lower ends of their assessment tool (e.g., Hendrix et al., 2020). Importantly, test developers should better define the characteristics of individuals with disabilities who are included and seek to include diverse samples of individuals with disabilities. Information about the normative sample composition is a critical part of assessment tool selection; therefore, the inclusion of this information would aid professionals when determining the best assessment tool for an individual client or student.

Until then, standardized, norm-referenced assessment tools that do not include individuals with disabilities broadly and/or NDDs specifically can still be used when working with this population. In particular, professionals can examine item-level performance and/or use growth or deviation scores to track change over time (e.g., Sansone et al., 2014). Professionals should also continue to use holistic approaches to assessment when working with individuals with NDDs by supplementing norm-referenced assessment tools with additional non-standardized assessment tools and dynamic assessment methods (Haywood and Tzuriel, 2002; Grigorenko, 2009).

### Study Limitations

There are several limitations to note in the current study. First, this review focused on normative samples, specifically. Many of the reported assessment tools have conducted follow-up validity or clinical research studies to test their measure on small groups of individuals with disabilities or NDDs. Although these participants are not included in the normative sample, the information can still be helpful for understanding if an assessment tool is appropriate for use with individuals with NDDs (e.g., if it will capture variability at lower ends, if items are appropriate, and/or if it yields valid and reliable scores in these populations). Future studies could review these validation studies to provide a comprehensive summary of the additional testing that has been conducted. Another limitation of the current study was that it excluded norm-referenced academic assessment tools that include a language subtest, as well as screeners and caregiver-, teacher-, and self-report measures. Therefore, we are unable to comment on their normative samples. Similarly, our review was limited to language, and we therefore cannot comment on norm-referenced measures of speech, other communication skills, or cognition more broadly. Lastly, although all discrepancies were resolved, we did not track the percentage of agreement across reviewers for the identification and coding of assessment tools and therefore cannot report inter-rater reliability.

## Conclusion

Researchers, clinicians, and educators who work with individuals with NDDs must often use standardized, norm-referenced language assessment tools. Unfortunately, many norm-referenced assessment tools have floor effects when used with individuals with intellectual disability or neurogenetic syndromes. We proposed that these floor effects may be due, in part, to the limited inclusion of individuals with NDDs in normative samples. However, even if some professionals wanted to use norm-referenced assessment tools that included individuals with NDDs in their normative samples, or at least that demonstrate variability at lower-performing ends of the assessment tool, this information can be difficult to access. Therefore, we reviewed and reported the representation of individuals with disabilities and NDDs in the normative samples of standardized, norm-referenced language assessment tools, as well as the range of standard/index scores provided. This information can be used to guide professionals’ selections of assessment tools, based on the individual or sample of individuals they are working with and the purpose of the assessment.

## Data Availability Statement

The original contributions presented in this study are included in the article/supplementary material, further inquiries can be directed to the corresponding author.

## Author Contributions

SL, MC, and LM conceptualized the study and drafted and edited the manuscript. SL and AB reviewed and coded assessments and drafted and edited tables for the Results section. All authors contributed to the article and approved the submitted version.

## Conflict of Interest

The authors declare that the research was conducted in the absence of any commercial or financial relationships that could be construed as a potential conflict of interest.

## Publisher’s Note

All claims expressed in this article are solely those of the authors and do not necessarily represent those of their affiliated organizations, or those of the publisher, the editors and the reviewers. Any product that may be evaluated in this article, or claim that may be made by its manufacturer, is not guaranteed or endorsed by the publisher.

## References

[B1] Abbeduto L. Berry-Kravis E. Sterling A. Sherman S. Edgin J. O. McDuffie A. (2020). Expressive language sampling as a source of outcome measures for treatment studies in fragile X syndrome: feasibility, practice effects, test-retest reliability, and construct validity. *J. Neurodev. Disord.* 12:10. 10.1186/s11689-020-09313-6 32204695 PMC7092603

[B2] Abbeduto L. McDuffie A. Thurman A. Kover S. T. (2016). Language development in individuals with intellectual and developmental disabilities: from phenotypes to treatments. *Int. Rev. Res. Dev. Disabil.* 50 71–118. 10.1016/bs.irrdd.2016.05.006

[B3] Abbeduto L. Thurman A. J. Bullard L. Nelson S. McDuffie A. (2019). “Genetic syndromes associated with intellectual disabilities,” in *Handbook of Medical Neuropsychology*, eds Armstrong C. Morrow L. (Cham: Springer), 263–299. 10.1007/978-3-030-14895-9_13

[B4] American Psychiatric Association [APA] (2013). *Neurodevelopmental Disorders Diagnostic and Statistical Manual of Mental Disorders: DSM-5*, Vol. 5. Washington, DC: American Psychiatric Association. 10.1176/appi.books.9780890425596

[B5] Bankson N. W. Bernthal J. E. (2020). *Bankson-Bernthal Test of Phonology*, 2nd Edn. Austin, TX: Pro-Ed, Inc. [Examiner’s Manual].

[B6] Bankson N. W. Mentis M. Jagielko J. R. (2018). *Bankson Expressive Language Test*, 3rd Edn. Austin, TX: Pro-Ed. [Examiner’s Manual].

[B7] Baumer N. T. Becker M. L. Capone G. T. Egan K. Fortea J. Handen B. L. (2022). Conducting clinical trials in persons with Down syndrome: summary from the NIH INCLUDE Down syndrome clinical trials readiness working group. *J. Neurodev. Disord.* 14:22. 10.1186/s11689-022-09435-z 35321660 PMC8942061

[B8] Berry-Kravis E. Hessl D. Abbeduto L. Reiss A. L. Beckel-Mitchener A. Urv T. K. (2013). Outcome measures for clinical trials in fragile X syndrome. *J. Dev. Behav. Pediatr.* 34 508–522. 10.1097/DBP.0b013e31829d1f20 24042082 PMC3784007

[B9] Blank M. Rose S. A. Berlin L. J. (2003). *Preschool Language Assessment Instrument*, 2nd Edn. Austin, TX: Pro-Ed. [Examiner’s Manual].

[B10] Bowers L. B. Huisingh R. LoGiudice C. (2006). *Listening Comprehension Test*, 2nd Edn. Austin, TX: Pro-Ed. [Examiner’s Manual].

[B11] Bowers L. B. Huisingh R. LoGiudice C. (2017a). *Social Language Development Test – Adolescent: Normative Update.* Austin, TX: Pro-Ed. [Examiner’s Manual].

[B12] Bowers L. B. Huisingh R. LoGiudice C. (2017b). *Social Language Development Test – Elementary: Normative Update.* Austin, TX: Pro-Ed. [Examiner’s Manual].

[B13] Bowers L. B. Huisingh R. LoGiudice C. Orman J. (2018b). *Expressive Language Test*, 2nd Edn Normative Update. Austin, TX: Pro-Ed. [Examiner’s Manual].

[B14] Bowers L. B. Huisingh R. LoGiudice C. (2018a). *Listening Comprehension Test – Adolescent: Normative Update.* Austin, TX: Pro-Ed. [Examiner’s Manual].

[B15] Bowers L. B. Huisingh R. LoGiudice C. Orman J. (2014). *The WORD Test: Elementary*, 3rd Edn. Austin, TX: Pro-Ed. [Examiner’s Manual].

[B16] Brady N. C. Fleming K. Romine R. S. Holbrook A. Muller K. Kasari C. (2018). Concurrent validity and reliability for the communication complexity scale. *Am. J. Speech Lang. Pathol.* 27 237–246. 10.1044/2017_AJSLP-17-0106 29383380 PMC5968331

[B17] Brady N. C. Fleming K. Thiemann-Bourque K. Olswang L. Dowden P. Saunders M. D. (2012). Development of the communication complexity scale. *Am. J. Speech Lang. Pathol.* 21 16–28. 10.1044/1058-0360(2011/10-0099) 22049404 PMC3273619

[B18] Brady N. C. Romine R. E. S. Holbrook A. Fleming K. K. Kasari C. (2020). Measuring change in the communication skills of children with autism spectrum disorder using the communication complexity scale. *Am. J. Intellect. Dev. Disabil.* 125 481–492. 10.1352/1944-7558-125.6.481 33211817

[B19] Budimirovic D. B. Berry-Kravis E. Erickson C. A. Hall S. S. Hessl D. Reiss A. L. (2017). Updated report on tools to measure outcomes of clinical trials in fragile X syndrome. *J. Neurodev. Disord.* 9:14. 10.1186/s11689-017-9193-x 28616097 PMC5467057

[B20] Carrow-Woolfolk E. (2011). *Oral and Written Language Scales*, 2nd Edn. Torrance, CA: Western Psychological Services. [Manual].

[B21] Carrow-Woolfolk E. (2014). *Test for Auditory Comprehension of Language*, 4th Edn. Austin, TX: Pro-Ed. [Examiner’s Manual].

[B22] Carrow-Woolfolk E. (2017). *Comprehensive Assessment of Spoken Language*, 2nd Edn. Torrance, CA: Western Psychological Services. [Manual].

[B23] Carrow-Woolfolk E. Allen E. A. (2014). *Test of Expressive Language.* Pro-Ed: Austin, TX. [Examiner’s Manual].

[B24] Carrow-Woolfolk E. Klein A. M. (2017). *Oral Passage Understanding Scale.* Bloomington, MN: NCS Pearson. [Examiner’s Manual].

[B25] Channell M. M. Loveall S. J. Conners F. A. Harvey D. J. Abbeduto L. (2018). Narrative language sampling in typical development: implications for clinical trials. *Am. J. Speech Lang. Pathol.* 27 123–135. 10.1044/2017_AJSLP-17-0046 29222570 PMC6105083

[B26] Dawson J. I. Stout C. E. Eyer J. A. (2003). *Structured Photographic Expressive Language Test*, 3rd Edn. Dekalb, IL: Janelle Publications. [Manual].

[B27] DiStefano C. Sadhwani A. Wheeler A. C. (2020). Comprehensive assessment of individuals with significant levels of intellectual disability: challenges, strategies, and future directions. *Am. J. Intellect. Dev. Disabil.* 125 434–448. 10.1352/1944-7558-125.6.434 33211812

[B28] Dodd B. Hua Z. Crosbie S. Holm A. Ozanne A. (2006). *Diagnostic Evaluation of Articulation and Phonology.* San Antonio, TX: Pearson Education.

[B29] Dunn D. M. (2019). *Peabody Picture Vocabulary Test*, 5th Edn. Bloomington, MN: NCS Pearson. [Manual].

[B30] Esbensen A. J. Hooper S. R. Fidler D. Hartley S. L. Edgin J. d’Ardhuy X. L. (2017). Outcome measures for clinical trials in Down syndrome. *Am. J. Intellect. Dev. Disabil.* 122 247–281. 10.1352/1944-7558-122.3.247 28452584 PMC5424621

[B31] Fudala J. B. Stegall S. (2017). *Arizona Articulation and Phonology Scale*, 4th Edn. Torrance, CA: Western Psychological Services. [Examiner’s Manual].

[B32] Gerhardstein Nader R. (2013). *Vocabulary Assessment Scales – Expressive & Receptive.* Lutz, FL: PARInc.

[B33] German D. J. (2014). *Test of Word Finding*, 3rd Edn. Austin, TX: Pro-Ed. [Examiner’s Manual].

[B34] Geurts H. M. Embrechts M. (2008). Language profiles in ASD, SLI, and ADHD. *J. Autism Dev. Disord.* 38 1931–1943. 10.1007/s10803-008-0587-1 18521730

[B35] Gilliam R. B. Pearson N. A. (2017). *Test of Narrative Language*, 2nd Edn. Austin, TX: Pro-Ed. [Examiner’s Manual].

[B36] Goldman R. Fristoe M. (2015). *Goldman-Fristoe Test of Articulation*, 3rd Edn. Bloomington, MN: NCS Pearson. [Manual].

[B37] Grigorenko E. L. (2009). Dynamic assessment and response to intervention: two sides of one coin. *J. Learn. Disabil.* 42 111–132. 10.1177/0022219408326207 19073895 PMC3575109

[B38] Hamaguchi P. Ross-Swain D. (2015). *Receptive, Expressive, and Social Communication Assessment - Elementary.* Novato, CA: Academic Therapy Publications. [Technical and Administration Manuals].

[B39] Hammill D. D. Brown V. Larsen S. Wiederholt L. (2007). *Test of Adolescent & Adult Language*, 4th Edn. Austin, TX: Pro-Ed. [Examiner’s Manual].

[B40] Hammill D. D. Newcomer P. L. (2019). *Test of Language Development – Intermediate*, 5th Edn. Austin, TX: Pro-Ed. [Examiner’s Manual].

[B41] Hawkins E. Gathercole S. Astle D., Calm Team, and Holmes J. (2016). Language problems and ADHD symptoms: how specific are the links? *Brain Sci.* 6:50. 10.3390/brainsci6040050 27775648 PMC5187564

[B42] Haywood H. C. Tzuriel D. (2002). Applications and challenges in dynamic assessment. *Peabody J. Educ.* 77 40–63. 10.1207/S15327930PJE7702_5 33486653

[B43] Helland W. A. Posserud M. B. Helland T. Heimann M. Lundervold A. J. (2016). Language impairments in children with ADHD and in children with reading disorder. *J. Atten. Disord.* 20 581–589. 10.1177/1087054712461530 23074303

[B44] Hendrix J. A. Amon A. Abbeduto L. Agiovlasitis S. Alsaied T. Anderson H. A. (2020). Opportunities, barriers, and recommendations in Down syndrome research. *Transl. Sci. Rare Dis.* 5, 99–129. 10.3233/TRD-200090 34268067 PMC8279178

[B45] Hessl D. Nguyen D. V. Green C. Chavez A. Tassone F. Hagerman R. J. (2009). A solution to limitations of cognitive testing in children with intellectual disabilities: the case of fragile X syndrome. *J. Neurodev. Disord.* 1 33–45. 10.1007/s11689-008-9001-8 19865612 PMC2768415

[B46] Hessling R. M. Schmidt T. J. Traxel N. M. (2004). “Floor effect,” in *Encyclopedia of Social Science Research Methods*, eds Lewis-Beck M. S. Bryman A. Liao T. F. T. (Thousand Oaks, CA: SAGE Publications, Inc), 390–391.

[B47] Hodson B. W. (2004). *Hodson Assessment of Phonological Patterns*, 3rd Edn. Austin, TX: Pro-Ed. [Examiner’s Manual].

[B48] Hresko W. Reid K. Hammill D. (2018). *Test of Early Language Development*, 4th Edn. Austin, TX: Pro-Ed. [Examiner’s Manual].

[B49] Huer M. B. Miller L. (2011). *Test of Early Communication and Emerging Language.* Austin, TX: Pro-Ed. [Examiner’s Manual].

[B50] Huisingh R. Bowers L. B. LoGiudice C. Orman J. (2002). *Test of Semantic Skills – Primary.* Austin, TX: Pro-Ed. [Examiner’s Manual].

[B51] Huisingh R. Bowers L. B. LoGiudice C. Orman J. (2019). *Test of Semantics Skills – Intermediate: Normative Update.* Austin, TX: Pro-Ed. [Examiner’s Manual].

[B52] Kasari C. Brady N. Lord C. Tager-Flusberg H. (2013). Assessing the minimally verbal school-aged child with autism spectrum disorder. *Autism Res.* 6 479–493. 10.1002/aur.1334 24353165 PMC4139180

[B53] Khan L. M. Lewis N. (2015). *Khan-Lewis Phonological Assessment*, 3rd Edn. Bloomington, MN: NCS Pearson. [Manual].

[B54] Lavi A. (2019). *Clinical Assessment of Pragmatics (CAPs).* Torrance, CA: Western Psychological Services. [Manual].

[B55] Lawrence B. Seifert D. (2016). *Test of Semantic Reasoning.* Novato, CA: Academic Therapy Publications. [Manual].

[B56] Leonard L. B. (1998). *Children with Specific Language Impairment.* Cambridge, MA: The MIT Press.

[B57] Luyster R. J. Seery A. Talbott M. R. Tager-Flusberg H. (2011). Identifying early-risk markers and developmental trajectories for language impairment in neurodevelopmental disorders. *Dev. Disabil. Res. Rev.* 17 151–159. 10.1002/ddrr.1109 23362034

[B58] Martin N. Brownell R. Hamaguchi P. (2018). *A Language Processing Skills Assessment*, 4th Edn. Novato, CA: Academic Therapy Publications. [Manual].

[B59] Martin N. A. (2013). *Expressive One-Word Picture Vocabulary Test*, 4th Edn Spanish-Bilingual. Novato, CA: Academic Therapy Publications. [Manual].

[B60] Martin N. A. Brownell R. (2011a). *Expressive One-Word Picture Vocabulary Test*, 4th Edn. Novato, CA: Academic Therapy Publications. [Examiner’s Manual].

[B61] Martin N. A. Brownell R. (2011b). *Receptive One-Word Picture Vocabulary Test*, 4th Edn. Novato, CA: Academic Therapy Publications. [Examiner’s Manual].

[B62] Mathews S. Miller L. (2015). *Test of Preschool Vocabulary.* Austin, TX: Pro-Ed. [Examiner’s Manual].

[B63] McBee M. (2010). Modeling outcomes with floor or ceiling effects: an introduction to the Tobit model. *Gifted Child Q.* 54 314–320. 10.1177/0016986210379095

[B64] McDuffie A. Thurman A. J. Channell M. M. Abbeduto L. (2017). “Language disorders in children with intellectual disability of genetic origin,” in *Handbook of Child Language Disorders*, 2nd Edn, ed. Schwartz R. (Milton Park: Taylor & Francis), 52–81. 10.4324/9781315283531-2

[B65] Montgomery J. (2008a). *Montgomery Assessment of Vocabulary Acquisition* (Expressive Vocab Test). Austin, TX: Pro-Ed. [Examiner’s Manual].

[B66] Montgomery J. (2008b). *Montgomery Assessment of Vocabulary Acquisition* (Receptive Vocab Test). Austin, TX: Pro-Ed. [Examiner’s Manual].

[B67] National Center for Education Statistics (2022). *Students with Disabilities. Condition of Education.* Washington, DC: U.S. Department of Education.

[B68] Nelson N. Plante E. Helm-Estabrooks N. Hotz G. (2016). *Test of Integrated Language and Literacy Skills.* Baltimore, MD: Brookes Publishing Company. [Examiner’s Manual].

[B69] Newcomer P. L. Hammill D. D. (2019). *Test of Language Development – Primary*, 5th Edn. Austin, TX: Pro-Ed. [Examiner’s Manual].

[B70] Peña E. D. Gutiérrez-Clellen V. F. Iglesias A. Goldstein B. A. Bedore L. M. (2018). *Bilingual English-Spanish Assessment.* Baltimore, MD: Brookes Publishing Company. [Test Manual].

[B71] Peña E. D. Spaulding T. J. Plante E. (2006). The composition of normative groups and diagnostic decision making: shooting ourselves in the foot. *Am. J. Speech Lang. Pathol.* 15 247–254. 10.1044/1058-0360(2006/023) 16896174

[B72] Phelps-Terasaki D. Phelps-Gunn T. (2007). *Test of Pragmatic Language*, 2nd Edn. Austin, TX: Pro-Ed. [Examiner’s Manual].

[B73] Reed R. (2018). “Toddlers and preschoolers with specific language impairment,” in *An Introduction to Children with Language Disorders*, 5th Edn, ed. Reed V. (New York, NY: Pearson Education), 77–129.

[B74] Richard G. J. Hanner M. A. (2005). *Language Processing Test 3: Elementary.* Austin, TX: Pro-Ed. [Examiner’s Manual].

[B75] Robertson C. Salter W. (2018). *Phonological Awareness Test*, 2nd Edn Normative Update. Austin, TX: Pro-Ed. [Examiner’s Manual].

[B76] Ross-Swain D. Long N. (2004). *Auditory Processing Abilities Test.* Novato, CA: Academic Therapy Publications. [Manual].

[B77] Sansone S. M. Schneider A. Bickel E. Berry-Kravis E. Prescott C. Hessl D. (2014). Improving IQ measurement in intellectual disabilities using true deviation from population norms. *J. Neurodev. Disord.* 6:16. 10.1186/1866-1955-6-16 26491488 PMC4613563

[B78] Schalock R. L. Luckasson R. Tassé M. J. (2021).? *Intellectual Disability: Definition, Diagnosis, Classification, and Systems of Supports.* Silver Spring: American Association on Intellectual and Developmental Disabilities. 10.1352/1944-7558-126.6.439 34700345

[B79] Secord W. A. Donohue J. S. (2014). *Clinical Assessment of Articulation and Phonology*, 2nd Edn. Austin, TX: Pro-Ed. [Examiner’s Manual].

[B80] Spaulding T. J. Plante E. Farinella K. A. (2006). Eligibility criteria for language impairment: is the low end of normal always appropriate? *Lang. Speech Hear. Serv. Schl.* 37 61–72. 10.1044/0161-1461(2006/007) 16615750

[B81] Straub L. Bateman B. T. Hernandez-Diaz S. York C. Lester B. Wisner K. L. (2022). Neurodevelopmental disorders among publicly or privately insured children in the United States. *JAMA Psychiatry* 79 232–242. 10.1001/jamapsychiatry.2021.3815 34985527 PMC8733868

[B82] Thurman A. J. Edgin J. O. Sherman S. L. Sterling A. McDuffie A. Berry-Kravis E. (2021). Spoken language outcome measures for treatment studies in Down syndrome: feasibility, practice effects, test-retest reliability, and construct validity of variables generated from expressive language sampling. *J. Neurodev. Disord.* 13:13. 10.1186/s11689-021-09361-6 33827417 PMC8028777

[B83] Tomblin J. B. Records N. L. Buckwalter P. Zhang X. Smith E. O’Brien M. (1997). Prevalence of specific language impairment in kindergarten children. *J. Speech Lang. Hear. Res.* 40, 1245–1260. 9430746 10.1044/jslhr.4006.1245 PMC5075245

[B84] Torgeson J. K. Bryant B. R. (2004). *Test of Phonological Awareness*, 2nd Edn Plus. Austin, TX: Pro-Ed. [Examiner’s Manual].

[B85] U.S. Department of Education (2000). *Annual Report to Congress on the Implementation of IDEA.* Washington, DC: U.S. Government Printing Office.

[B86] Van Herwegen J. Riby D. Farran E. K. (2015). “Neurodevelopmental disorders: definitions and issues,” in *Neurodevelopmental Disorders: Research Challenges and Solutions*, eds Van Herwegen J. Riby D. (London: Psychology Press), 3–18. 10.4324/9781315735313

[B87] Wagner R. K. Torgesen J. K. Rashotte C. A. Pearson N. A. (2013). *Comprehensive Test of Phonological Processing*, 2nd Edn. Austin, TX: Pro-Ed. [Examiner’s Manual]. 10.1037/t52630-000

[B88] Wallace G. Hammill D. D. (2013). *Comprehensive Receptive and Expressive Vocabulary Test*, 3rd Edn. Austin, TX: Pro-Ed. [Examiner’s Manual].

[B89] Wetherby A. M. Prizant B. M. (2002). *Communication and Symbolic Behavior Scales, Normed Edition.* Baltimore, MD: Brookes Publishing Company. [Manual]. 10.1037/t11527-000

[B90] Wiig E. H. Secord W. A. (2006). *Emerging Literacy & Language Assessment.* Greenville, SC: Super Duper Publications. [Manual].

[B91] Wiig E. H. Secord W. A. Semel E. (2020). *Clinical Evaluation of Language Fundamentals Preschool*, 3rd Edn. Bloomington, MN: NCS Pearson. [Manual].

[B92] Wiig E. H. Semel E. Secord W. A. (2013). *Clinical Evaluation of Language Fundamentals*, 5th Edn. Bloomington, MN: NCS Pearson. [Manual].

[B93] Williams K. T. (2014). *Phonological and Print Awareness Scale.* Torrance, CA: Western Psychological Services. [Manual].

[B94] Williams K. T. (2018). *Expressive Vocabulary Test*, 3rd Edn. Bloomington, MN: NCS Pearson. [Manual].

[B95] World Health Organization [WHO] (2019). *International Classification of Diseases for Mortality and Morbidity Statistics (11th Revision).* Geneva: World Health Organization.

[B96] Young N. A. E. (2021). *“Childhood Disability in the United States: 2019,” ACSBR-006, American Community Survey Briefs.* Washington, DC: U.S. Census Bureau.

[B97] Zhu L. Gonzalez J. (2017). Modeling floor effects in standardized vocabulary test scores in a sample of low ses Hispanic preschool children under the multilevel structural equation modeling framework. *Front. Psychol.* 8:2146. 10.3389/fpsyg.2017.02146 29312033 PMC5732956

[B98] Zimmerman I. Steiner V. Pond R. (2011). *Preschool Language Scales*, 5th Edn. Bloomington, MN: NCS Pearson. [Manual]. 10.1037/t15141-000

